# Genome-scale metabolic network reconstructions of diverse *Escherichia* strains reveal strain-specific adaptations

**DOI:** 10.1098/rstb.2021.0236

**Published:** 2022-10-10

**Authors:** Jonathan M. Monk

**Affiliations:** Department of Bioengineering, University of California, 9500 Gilman Drive, San Diego, La Jolla, CA 92093-0412, USA

**Keywords:** genome-scale modelling, *Escherichia*, metabolic network reconstruction

## Abstract

Bottom-up approaches to systems biology rely on constructing a mechanistic basis for the biochemical and genetic processes that underlie cellular functions. Genome-scale network reconstructions of metabolism are built from all known metabolic reactions and metabolic genes in a target organism. A network reconstruction can be converted into a mathematical format and thus lend itself to mathematical analysis. Genome-scale models (GEMs) of metabolism enable a systems approach to characterize the pan and core metabolic capabilities of the *Escherichia* genus. In this work, GEMs were constructed for 222 representative strains of *Escherichia* across HC1100 levels spanning the known *Escherichia* phylogeny. The models were used to study *Escherichia* metabolic diversity and speciation on a large scale. The results show that unique strain-specific metabolic capabilities correspond to different species and nutrient niches. This work is a first step towards a curated reconstruction of pan-*Escherichia* metabolism.

This article is part of a discussion meeting issue ‘Genomic population structures of microbial pathogens’.

## 1. Introduction

*Escherichia coli* K-12 MG1655 is the model organism for research on microbial metabolic systems biology and physiology [1]. Recent studies have demonstrated that this strain is not representative of the diversity of metabolic capabilities across the species [2,3]. Genome-scale models (GEMs) of metabolism for hundreds of strains in this species have estimated the *E. coli* ‘core’ metabolic reactome to be 1866 reactions [4]. However, work reconstructing *E. coli* metabolism has been focused on available genome sequences that have not always spanned the known *Escherichia* phylogeny.

EnteroBase represents a curated database of over 100 000 *E. coli* strains (191 199 strains as of 1 November 2021) [5]. Clustering methods have divided these strains into groups that span the currently known *Escherichia* phylogeny. EnteroBase automatically clusters core genome multi-locus sequence typing (MLST) allelic profiles into hierarchical clusters (HierCC) from annotated genomes. EnteroBase has implemented HierCC for core genome MLST, which allows for resolution of population structures at multiple levels ranging from HC2000 (super lineages) for intercontinental dispersion down to HC5-10 for detecting local transmission chains. EnteroBase reports cluster assignments and designations at 11 levels of allelic differences for the *Escherichia* genus*.* HierCC is able to assign genomes to populations and lineages within *Escherichia/Shigella*, and compares favourably with other methods such as MLST and average nucleotide identity (ANI) [6]. Thus, HierCC can be used to identify representative strains of *Escherichia* spanning the diversity across the genus. Metabolic network reconstructions have demonstrated their use to computationally evaluate the genomic diversity of metabolism between organisms [7,8]. This study aimed to take a first step towards reconstructing metabolic network reconstructions and GEMs of metabolism for a set of strains spanning the *Escherichia* phylogeny.

### (a) Strain selection

**Figure 1.**
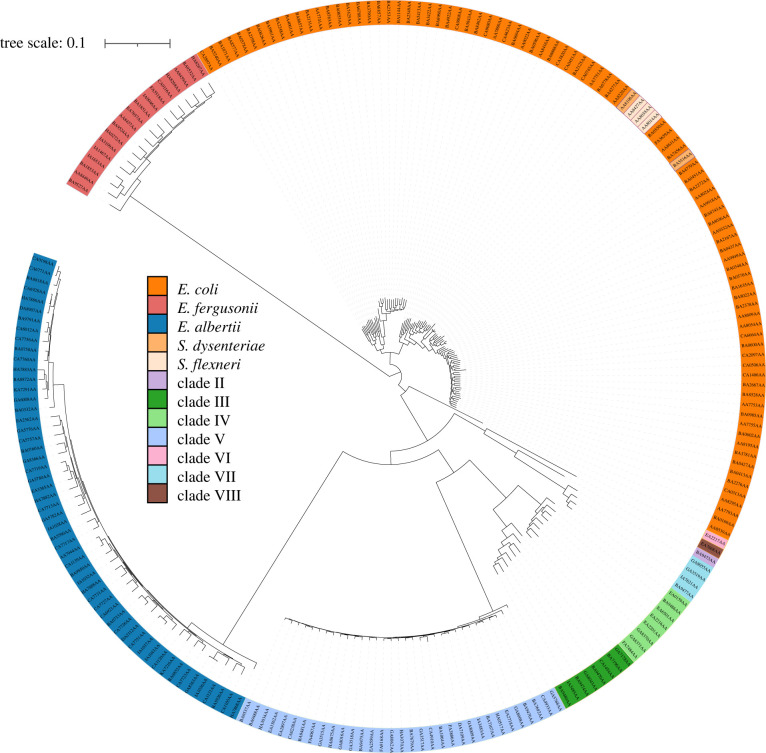
SNP tree of all 222 *Escherichia* strains spanning 222 distinct HC1100 clusters. Tree is based on SNPs found within the core-genome of all strains. The 222 strains spanned 12 diverse taxonomic groupings with an average of 20 ± 27 strains per lineage.

**Figure 2.**
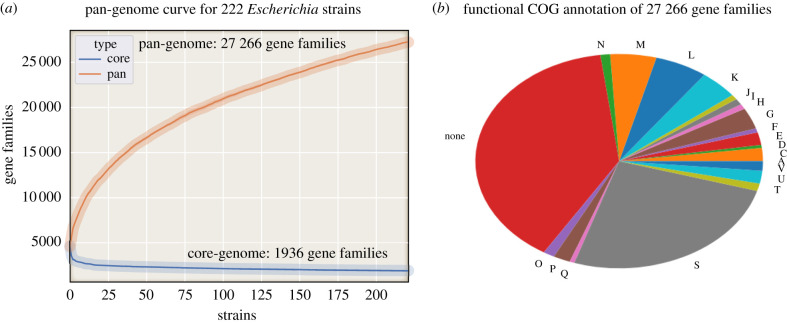
Pan-genome analysis of the 222 representative strains. (*a*) Pan-genome curve representing the number of shared (core) genes and unique (pan) genes counted as additional strains are added (*x*-axis). Strains were added in random order 10 times with differences displayed as shaded curves representing 95% confidence intervals. (*b*) Functional clusters of orthologous group (COG) annotation of the pan-genome. Abbreviations: A, RNA processing and modification; C, energy production and conversion; D, cell cycle control; E, amino acid metabolism and transport; F, nucleotide metabolism and transport; G, carbohydrate metabolism and transport; H, coenzyme metabolism; I, lipid metabolism; J, translation; K, transcription; L, replication and repair; M, cell wall/membrane/envelope biogenesis; N, cell motility; O, post-translational modification, protein turnover, chaperone functions; P, inorganic ion transport and metabolism; Q, secondary metabolites biosynthesis, transport and catabolism; T, signal transduction; U, intracellular trafficking and secretion; V, defence mechanisms; S, function unknown.

A total of 222 genome sequences were selected from the over 100 000 available on Enterobase using hierarchical clustering of core genome sequence types with the EnteroBase HierCC pipeline. The genomes were selected based on their diversity spanning the *Escherichia* phylogeny (figs 1 and 2 in companion manuscript [6] for a tree of 967 genomes representing the known core genome diversity of Escherichia in 2021). HierCC assignments were more consistent with maximum-likelihood super-trees of core single nucleotide polymorphisms (SNPs) or presence/absence of accessory genes than classical taxonomic assignments or 95% ANI [6]. Thus, HC1100 groups are a good tool for detecting populations within *Escherichia*. Strain names, assembly data and hierarchical clustering (HC) groups are available in the electronic supplementary material, data file S1. Beyond *Escherichia coli*, the *Escherichia* genus also includes species *albertii*, *fergusonii*, *marmotae* and *ruysiae*. Furthermore, common causes of dysentery *Shigella boydii*, *Shigella dysenteriae*, *Shigella flexneri* and *Shigella sonnei* all correspond to phylogenetic lineages within *E. coli* rather than to discrete taxonomic units. The 222 strains collected span the *Escherichia* phylogeny across 12 clades including strains from clades I–VII as well as representatives of *Escherichia fergusonii* (*n* = 11), *Escherichia albertii* (*n* = 54) and *Shigella* (*n* = 5) (figure 1). All strains were determined to be from distinct sequence types as defined by MLST [9].

### (b) Pan-genome analysis

The 222 strains were used to construct a pan-genome to evaluate shared and unique genes between the strains [10]. There were a total of 27 266 unique gene families present across the 222 strains of which 1936 were shared by all 222 strains (core genome). eggNOG [11] was used to functionally annotate representative amino acid sequences from each of the 27 266 gene families (figure 2*a*). eggNOG failed to assign functions to 10 669 (39.1%) genes and another 6967 (25.6%) were annotated as having ‘unknown function’ (figure 2*b*). Thus, in total, 64.7% of genes in the pan-genome were functionally unannotated. For those genes where functional annotation was available the top five categories included 535 (2.0%) in energy production and conversion, 867 (3.2%) in carbohydrate metabolism and transport, 1181 (4.3%) in transcription, 1414 (5.2%) in cell wall/membrane/envelope biogenesis and 1661 (6.1%) in replication and repair. A full pan-genome presence/absence matrix with annotated functions is available in the electronic supplementary material, data file S4.

### (c) Characteristics of the core and pan models

**Figure 3.**
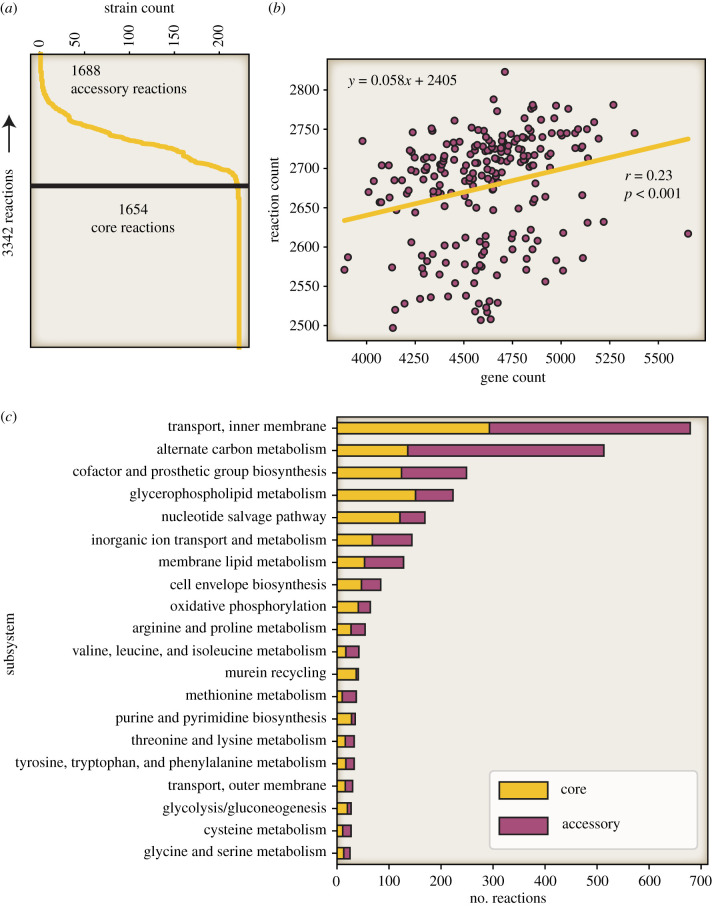
Graphical representation of core and pan reactomes. (*a*) The total metabolic reactome consisted of 3342 reactions; 1654 of these were shared by all 222 strains representing the core reactome. By contrast, 1668 reactions were found in a subset of the 222 strains as represented by the *x*-axis. (*b*) The number of genes in each strain plotted against the number of reactions in each strain-specific model. A low level of correlation (Pearson *r* = 0.23, *p* < 0.005) was observed between gene count and number of model reactions fitting a line described as *y* (number of model reactions) = 0.058 × (number of genes) + 2405. (*c*) The distribution of core and accessory reactions across metabolic subsystems.

A set of 222 *E. coli* genome-scale reconstructions were built by combining two recently published workflows [12,13] and used to compare gene, reaction and metabolite content. The content shared among all reconstructions defines the ‘core’ metabolic capabilities among all the strains. Similarly, the metabolic capabilities of all the strains were combined to define the full set that encompasses all models and thereby define the ‘pan’ metabolic capabilities among all the strains (figure 3*a*). The GEMs covered 3393 out of the 27 266 gene-families present in the calculated pan-genome (12.4%).

The size and content of the core metabolic content can be used to characterize the metabolic basis of *E. coli* as a species. There were 1688 reactions shared across all strains. The reactions in the core group fell into specific metabolic subsystems including the pentose phosphate pathway (12 out of 13 reactions, 92% conserved), murein biosynthesis and recycling (52 out of 59 reactions, 88% conserved), purine and pyrimidine metabolism (28 out of 35 reactions, 80% conserved) and (glycolysis/gluconeogenesis (20 out of 27 reactions, 74% conserved). Furthermore, some of the reactions not classified as core reactions were still found in a majority of strains, for example in glycolysis/gluconeogenesis the glucose-1-phosphate adenylyltransferase reaction encoded for by *glgC* was found in all but one strain. See the electronic supplementary material, data file S2 for full details.

By contrast with the core reconstruction, the pan metabolic content constitutes the total number of different reactions found in all strains and as such is an indicator of the full metabolic capabilities within the *Escherichia* genus. There were a total of 3342 reactions found in any strain, of these 1688 were variably present across the strain-specific models. The model with the most reactions was *E. coli* AZ-TG73683 (2823 reactions) while the model for *E. albertii* O88:H- had the fewest number of reactions (2497 reactions) (figure 3*b*).

By contrast to subsystems common to core reactions, the accessory reactome was found to have reactions from nitrogen metabolism (5 out of 23 reactions, 22% conserved), alternative carbon metabolism (136 out of 513 reactions, 27% conserved) and for the amino acid methionine (10 out of 27 reactions, 27% conserved). Importantly, the alternate carbon metabolism subsystem is by far the largest of these groups (513 reactions).

### (d) Calculating phenotypes

Metabolic network reconstructions can be converted to computable mathematical models allowing them to compute phenotypes (outputs) given different inputs [14,15]. The 222 strain-specific reconstructions were converted to mathematical models allowing simulation of growth in different environments including all possible sets of potentially growth supporting carbon, nitrogen, phosphorus and sulfur sources. Thus, this set of GEMs allows for a meaningful interpretation of the content of each reconstruction and allows one to gain perspective on the strain's micro-environmental and ecological niche [16].

**Figure 4.**
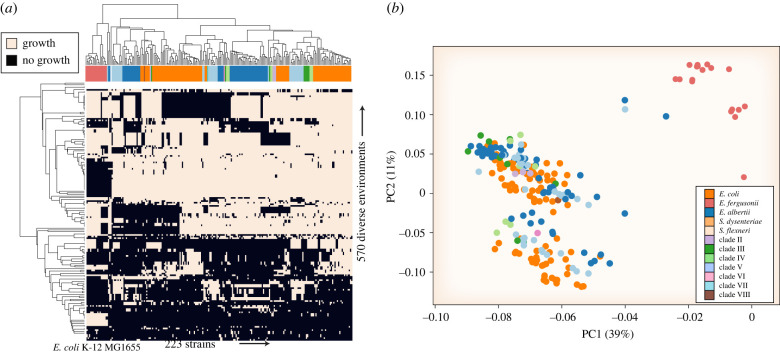
Model-predicted growth capabilities in 570 different growth-supporting nutritional environments. Growth environments were composed of alternate carbon, nitrogen, phosphorus and sulfur sources). (*a*) Clustered heatmap of predicted growth is represented by black and no-growth is represented by white. The taxonomic designation for each strain is represented by the horizontal bar at the top of the heatmap with colours corresponding to the legend in panel (*b*). The common laboratory strain *E. coli* K-12 MG1655 was included for context. (*b*) Principal component analysis (PCA) plot of strains based on predicted growth capabilities. Full growth predictions are available in the electronic supplementary material, data file S3.

Previous work has shown that alternate carbon sources distinguish strains [17,18]. Thus, simulations were performed to predict growth capabilities in alternate environments including sole growth supporting carbon, phosphorus and nitrogen sources. In total, 735 different growth conditions were evaluated. At least one strain was able to grow in 570 unique environments. Of these, 220 supported growth for all strain-specific models (figure 4*a*) (electronic supplementary material, data file S3).

We observed variation in catabolic capabilities across the HC1100 representatives. This result aligns with observations of variable O-antigens presence throughout *Escherichia* [6] and further bolsters observations regarding the high frequency of homologous recombination in this genus [19,20]. Some of the most variable growth-supporting carbon sources included 4-hydroxyphenylacetate (74% models predicted to grow), rhamnose (73%), myo-inositol (61%), (R)-propane-1,2-diol (48%) and allose (34%) (figure 4*b*). Some of the fewest number of strains were predicted to grow on d-xylonate as a carbon source (5% of strains), d-lysine (7%) and l-fuculose (3%). Variable sole nitrogen source compounds predicted to support growth included d-ornithine (8% of strains), psicoselysine (29%), acetamide (72%) and d-methionine (82%). l-cysteate was one of the compounds predicted to support growth as a sole nitrogen source across the fewest number of strains (3%). Phosphorus sources were far more conserved with the majority of compounds (50 out of 60) predicted to support growth in greater than 95% of strains. The most variable predicted growth supporting phosphorus sources were Arbutin 6-phosphate (59% of strains predicted to grow) and 2-phosphoglycolate (72%). l-cysteate was among the least predicted sole sulfur sources to support growth (2% of strains), while compounds like taurine (69%) and isethionic acid (69%) had more variable predictions to support growth as sole sulfur sources.

## 2. Discussion

This study demonstrates the use of GEMs of metabolism to study similarities and differences between species and strains of a genus. Unique GEMs for 222 different *Escherichia* strains spanning the phylogeny were constructed and used to: (i) tabulate core and pan metabolic capabilities within the *Escherichia* genus; and (ii) calculate metabolic capabilities and evaluate differences between strains by computing growth phenotypes on over 500 different nutrients. This work further bolsters the case for using strain-specific models of *Escherichia* to guide future studies to evaluate growth advantages conferred by unique nutrients to *Escherichia* strains in different niches [21]. All in all, this study begins the process of defining the *Escherichia* genus based on common metabolic capabilities, and its strains based on niche-specific growth capabilities.

The results obtained generate new hypotheses related to a strain's nutrient niche. Only 7% of strains were predicted to catabolize the sugar acid d-xylonate as a carbon source. This may indicate *Escherichia* strains seldom encounter this compound. d-xylonate is derived from the hemicellulose sugar d-xylose and has several industrial applications [22]. Two natural pentitols ribitol and d-arabitol were predicted to be catabolized by 13% of strains. Thus, strains that catabolize these compounds may be favoured in niches where they consist as part of the diet. For example, d-arabitol occurs naturally in certain forms of mushrooms (at up to 9.5% of dry weight) and ribitol occurs bound to the teichoic acids and capsules of several Gram-positive bacteria [23].

Another interesting carbon source is d-allose where only 34% of strains were predicted to catabolize this compound. d-allose is a monosaccharide rarely found in the natural environment but touted as a potential ultra-low calorie sweetener [24]. Our results indicate that some strains of *Escherichia* may gain a fitness advantage compared to other strains from catabolism of d-allose and thus further studies should be performed to evaluate the potential impact to the microbiome upon ingestion of d-allose. Similarly, tagatose is another hexose often used as an artificial sweetener. Our models predicted 39% of the strains were able to catabolize this compound as a sole carbon source.

Beyond carbon sources, d-ornithine was predicted to support growth as both a sole nitrogen and carbon source for 8% of strains. d-ornithine has been detected in cow milk and thus may form a source of both carbon and nitrogen for strains present in environments rich in dairy products. Taurine was predicted to serve as a sole sulfur source for 69% of strains. Taurine is a major component of bile and has been detected in the large intestine. It is also a common ingredient in energy drinks.

The core reactome identified in this study consisted of 1688 reactions, a count slightly smaller (1866) than a recent study looking solely at *E. coli* strains [4]. This is probably owing to the inclusion of other species in the *Escherichia* genus such as *E. fergusonii* and *E. albertii.* It should also be noted that these models were built based on a database of metabolic enzymes present in Gram-negative species. Functional characterization of enzymes in diverse strains is constantly improving and thus these models should be viewed as a work in progress. Furthermore, it is possible that more distantly related species have orthologous proteins with greater amino acid differences. Future work should include a deeper analysis of the orthology cut-offs used for model construction and should aim to evaluate the sequence-level diversity of functionally similar enzymes across these more distantly related species.

Minimal gap-filling was performed on these models to ensure they could grow in M9-minimal media with glucose as the carbon source. As a result, we expect these models to faithfully capture the diverse catabolic capabilities of the different strains. However, strain-specific auxotrophies may be obfuscated by this gap-filling. Future work could focus on delineating differences in strain-specific auxotrophy by limiting gap-filling and evaluating missing ‘black holes’ in anabolic pathways [25,26]. Because the reconstruction process is iterative, comparing the model predictions generated here with experimental growth screens would highlight areas where the model predictions are incorrect and would guide further curation and improvements [27].

This work is the first step towards a pan-metabolic reconstruction of the *Escherichia* genus. Further literature curation and experiments will be required. The reconstruction process is iterative and thus testing of model-predicted phenotypes is essential. Strain acquisition can be difficult, however collecting these strains for high-throughput phenotypic screens (e.g. BioLog [28]) would be useful to improve these models and guide further curation and validation. A highly curated reconstruction of *Escherichia* metabolic capabilities would be a valuable resource to the community of systems modellers and those studying *Escherichia* physiology. It would allow for deeper elucidation of the genotype to phenotype relationship across diverse strains of the genus. Furthermore, such a resource would allow for rapid, high-quality construction of strain-specific models of freshly acquired and sequenced isolates.

## 3. Material and methods

### (a) Strain specific model reconstruction

All genomes were re-annotated using the PROKKA v. 1.12 [29]. Amino acid sequences from *E. coli* K-12 MG1655 were used for identifying orthologues following the protocol by Norsigian *et al.* [12] using a bi-directional hit cut-off of 70% over at least 70% of the protein length. CarveME [13] was used to supplement these reconstructions using a database of Gram-negative metabolic reconstructions (-u gramneg option). The models were gap-filled using M9 minimal media (-g M9 option). MetaNetX [30] was used to standardize metabolites and reactions to the BiGG (Biochemical Genetic and Genomic knowledgebase) namespace [31]. All genome sequences were downloaded from EnteroBase on 11 February 2020.

### (b) *In silico* growth simulations

The COBRApy toolbox v. 0.22.1 [32] was used for all simulations. Each of the 223 metabolic network reconstructions (including *E. coli* K-12 MG1655 model iML1515) were loaded into the toolbox. M9 minimal media was simulated by setting a lower bound of −1000 (allowing unlimited uptake) on the exchange reactions for Ca^2+^, Cl^−^, CO_2_, Co^2+^, Cu^2+^, Fe^2+^, Fe^3+^, H^+^, H_2_O, K^+^, Mg^2+^, Mn^2+^, ${\mathrm{MoO}}_{4}^{2-}$, Na^+^, Ni^2+^, ${\mathrm{SeO}}_{4}^{2-}$, ${\mathrm{SeO}}_{3}^{2}$ and Zn^2+^. A lower bound of −0.01 was placed on the cob(I)alamin exchange reaction. The default carbon source was glucose with a lower bound of −20, the default nitrogen source was NH_4_^−^ with a lower bound of −1000, the default phosphorus source was ${\mathrm{HPO}}_{4}^{2}$ with a default bound of −1000 and the default sulfur source was ${\mathrm{SO}}_{4}^{2-}$ with a default bound of −1000. To identify sole growth supporting carbon, nitrogen, phosphorus and sulfur sources each of these default compounds were removed from the media (lower bound set to 0) one at a time and different compounds were added to determine whether they supported growth. All simulations were performed in aerobic conditions with O_2_ added with a lower bound of −20. Nutrient sources with growth rates above zero were classified as growth supporting, while nutrient sources with growth rates of zero were classified as non-growth supporting. The Gurobi 9.1.2 linear programming solver (Gurobi Optimization Inc., Houston, TX) was used to perform flux-balance analysis.

### (c) Heatmap, phylogenetic tree, pan-genome and principal component analysis figure construction

The pan-genome and core-genome SNP tree was constructed by calculating the core-genome using panX [10]. A core-genome SNP matrix was constructed to build the core-genome phylogenetic tree using FastTree [33] and RaxML [34]. Genes of the pan-genome were annotated using eggNOG v. 5.0 [11]. The binary results from the growth/no growth simulations for each strain were used to compute a hierarchical clustering using the Jaccard method in the seaborn python package. The heat map was visualized using matplotlib in python. The principal component analysis plot was built from predicted growth values using the scikit-learn implementation [35].

## Data Availability

All data are accessible as part of the electronic supplementary material [36].
